# Giant Cell Tumour of Bone: A Comprehensive Review of Pathogenesis, Diagnosis, and Treatment

**DOI:** 10.7759/cureus.46945

**Published:** 2023-10-13

**Authors:** Yash Jha, Kirti Chaudhary

**Affiliations:** 1 Anatomy, Jawaharlal Nehru Medical College, Datta Meghe Institute of Higher Education and Research, Wardha, IND

**Keywords:** imaging of giant cell tumor, grading of gaint cell tumor of bone, treatment of gaint cell tumor, diagnosis of gaint cell tumor of bone, giant cell tumor of bone (gctb)

## Abstract

The benign aggressive tumour known as a giant cell tumour of bone (GCTB) frequently affects the knee bones. Patients suffering from GCTB present with pain, swelling, joint effusion, loss of ability to bear weight on the involved extremity and a restriction in the range of motion of the afflicted joint may also exist, depending on the tumour's size. GCTB makes up 20% of benign skeletal tumours and 5% of all primary bone tumours. Although it has an equal distribution of the sexes, the majority reveal a higher frequency among women. Eighty per cent of GCTB instances were recorded in patients between the ages of 20 and 50 during the third decade. The femur, tibia and radius are where GCTB is most frequently discovered. Lesions can be rated using the Campanacci grading method based on the plain radiograph's results. Plain radiography, CT and MRI are used to diagnose the tumour. Surgery is the only curative treatment which is determined by the Campanacci grade and the tumour's location. Recurrence of GCTB is observed in about 25% of patients, with curettage being associated with rates as high as 50%. We evaluated the GCTB-related articles and summarised the developments in diagnosis, treatment and reducing risk of recurrence.

## Introduction and background

Giant cell tumour of bone (GCTB), first described in 1818, is a benign primary bone tumour marked by multinuclear giant cells and has a high propensity to return locally after treatment [1]. It can occasionally change into a malignant version. Ten per cent of cases are diagnosed within the second decade of life when multicentric tumours and spine tumours are more common. These massive cells are not the true neoplastic cells, but they do cause bone resorption. The osteolytic lesion that is characteristic of this tumour is thought to be formed by multinucleated "giant cell" development from the stromal cells, which are thought to be the true neoplastic cells [2]. Neoplastic mononuclear stromal cells with spindled fibroblast-like shapes and reactive round or multinucleated giant cells with an osteoclast-like morphology make up GCTB. The following are indicators from research showing the mononuclear stromal cells are actual neoplastic components - mononuclear stromal cells are more prevalent in GCTB than multinucleated large cells, and they also exhibit greater proliferative capability, genetic abnormalities, and expression of key cytokines and differentiation markers [3].

It is uncommon for GCT to involve the metatarsal bone in patients with underdeveloped skeletons [4]. It is also uncommon for GCT to be seen in the little bones of the hand and foot. The primary lesion is typically 2.9 x 2.6 x 2.6 centimetres in size [5]. GCTs of the soft tissue have a lower local recurrence rate than GCTs of the bone, but they are more likely to spread and cause death [6]. In comparison to other common aggressive diseases, GCTB does not exhibit any distinguishing clinical or imaging characteristics of malignancy. The prognosis for malignant GCTs of the bone has historically been bad, and it gets worse when secondary malignancies are present [7]. A total body CT scan of an adult female who was partially mummified and dated to the eighteenth century revealed GCT of the left femur [8]. Another benign bone tumour that is rich in giant cells is known as central giant cell granuloma (CGCG), which is thought to be the outcome of a local reparative reaction in most people between the ages of 10 and 25 [9].

## Review

Epidemiology

GCT makes up 20% of benign skeletal tumours and 5% of all primary bone tumours. Southern India and China, where GCT accounts for 20% of all primary bone tumours, have an especially high prevalence. Although some studies have claimed an equal distribution of the sexes, the majority reveal a higher frequency among women. Eighty per cent of GCT affects patients between the ages of 20 and 50 during the third decade when the prevalence of the condition increases. Less than 3% of cases include children under the age of 14, and only 13% involve patients over the age of 50. Most lesions (75%-90%) form in long bones, with the knee accounting for the bulk of cases (50%-65%) [10]. Paget disease may co-occur with GCT, which most frequently affects the skull, face bones, pelvis, and spine [10].

Site

Although GCTB can develop anywhere in the skeleton, it usually manifests as an extension of the metaphysis into the epiphysis in long bones [2]. The distal femur, proximal tibia, and distal radius are where GCT is most frequently discovered. The sacrum is where GCT is most frequently found in the axial spine. Rarely do the posterior elements and the vertebral body of the mobile spine contain GCT [1]. Head and neck GCTs account for 2% of all GCTs. The sphenoid, ethmoid, or temporal bones are where GCTs in the head and neck region most frequently develop. The hyoid bone and the cartilaginous framework of the larynx are only very infrequently affected [11]. In 1940, Wessely published the first example of a laryngeal GCT. Larynx GCTs are benign tumours with a reportedly good cure rate [11].

Grading system

The tumour often shows up as an irregular region of bone loss on conventional radiography. In cases of a higher grade, the cortex may thin and perforate. Lesions can be rated using the Campanacci grading method based on the plain radiograph's results. Lesions of Grade I are limited to the bone. Grade II lesions are those that feature an enlargement of the cortex but no perforation and Grade III lesions are those that have soft tissue extension and cortical perforation [2]. The difference between Enneking and Campanacci systems is mentioned in Table 1 [1].

**Table 1:** Comparison of bone tumour grading systems using Enneking and Campanacci

Grade	Enneking System	Campanacci System
1	Benign, indolent, or biologically static	Radiographically well-circumscribed lucent lesion with no aggressive features (eg, periosteal reaction, soft-tissue mass, cortical breach). Rare.
2	Progressive growth, limited by natural barriers	Relatively well-defined radiographic borders without a radiopaque rim.
3	Locally aggressive with corresponding soft-tissue mass	Indistinct or ill-defined borders with radiographic demonstration of cortical bone destruction, and a soft-tissue mass [1].

Symptoms

Pain is the most typical GCTB-presenting symptom. Additional symptoms include swelling, joint effusion, and a restriction in the range of motion of the afflicted joint may also exist, depending on the tumour's size. Some symptoms, including neurological symptoms in GCTB of the axial skeleton, are specific to the location of the tumour. When the cortex of weight-bearing bones thins, patients may even present with pathological fractures [2].

Imaging

A physical examination is done after getting a patient's medical history. Plain radiography, computed tomography (CT), and magnetic resonance imaging (MRI) are used to assess the main tumour. While plain radiography and CT are helpful for assessing the tumour's bone component, MRI is better suited for evaluating the tumour's intramedullary and soft tissue expansion. Finding pulmonary metastases, which may exist in a very tiny proportion of patients, requires the use of a chest radiograph. To find more skeletal illness areas, bone scans are helpful [12]. The "donut sign" is a common manifestation of the scintigraphic features of GCTB, which include strong radionuclear uptake in the peripheral due to hyperemia and photopenia in the centre due to necrosis. For the purpose of looking for potential lung metastases, chest imaging is necessary [13]. According to the tumour's growth pace, which varies from patient to patient, imaging of the bone suggests the diagnosis of GCT when there is uniform bone lysis, no mineralized signal, metaphyso-epiphyseal, well limited without osteosclerotic border, or somewhat blurred. Typically, the lysis is eccentric. The cortex is frequently "blown out" and distorted [14].

**Figure 1:**
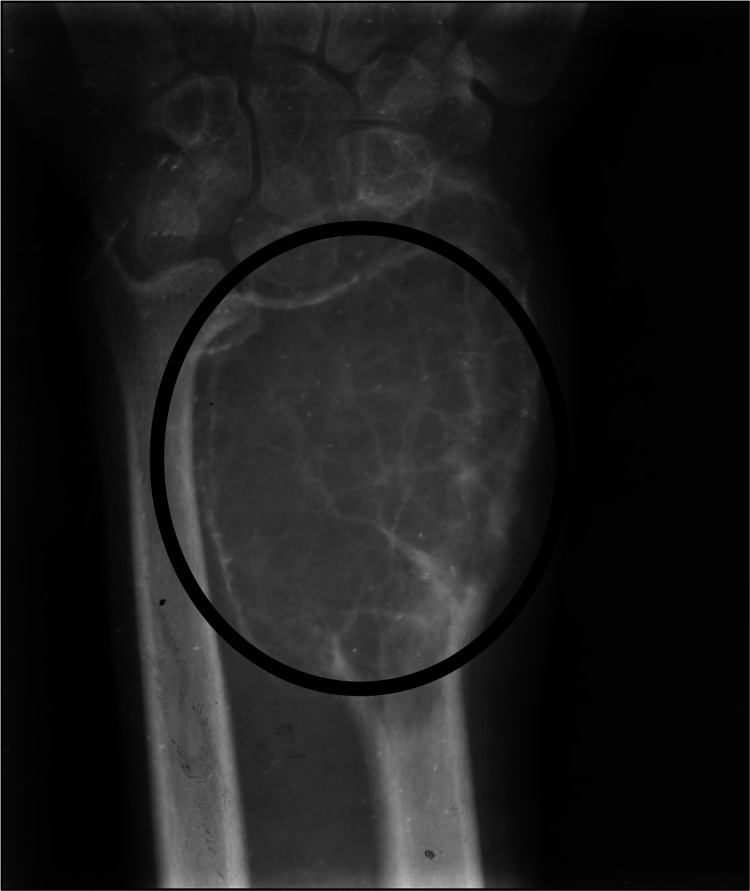
X-ray of wrist joint showing GCTB in the lower end of radius. Image taken by Yash Jha. GCTB: giant cell tumour of bone

**Figure 2:**
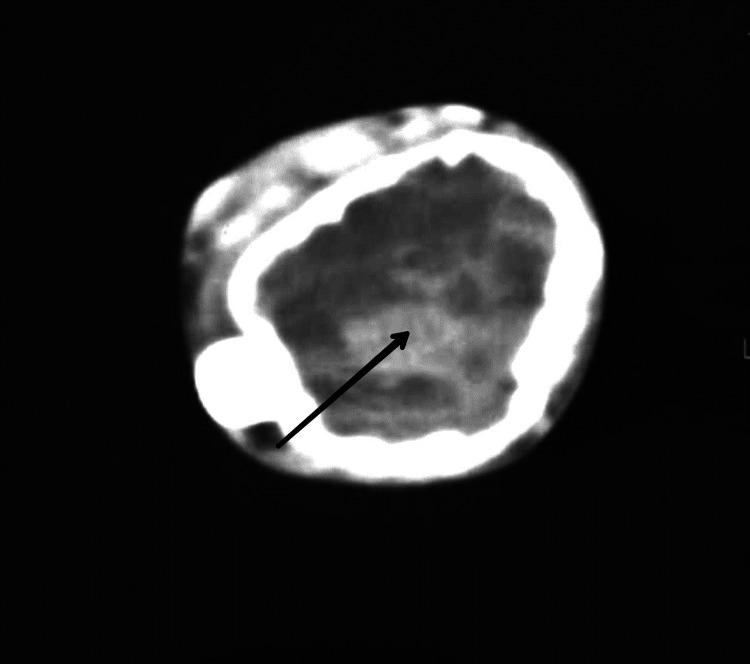
CT of wrist joint showing GCTB in the lower end of radius extending in the subarticular region with a thin zone of transition and without a cortical break. Image taken by Yash Jha. GCTB: giant cell tumour of bone

**Figure 3:**
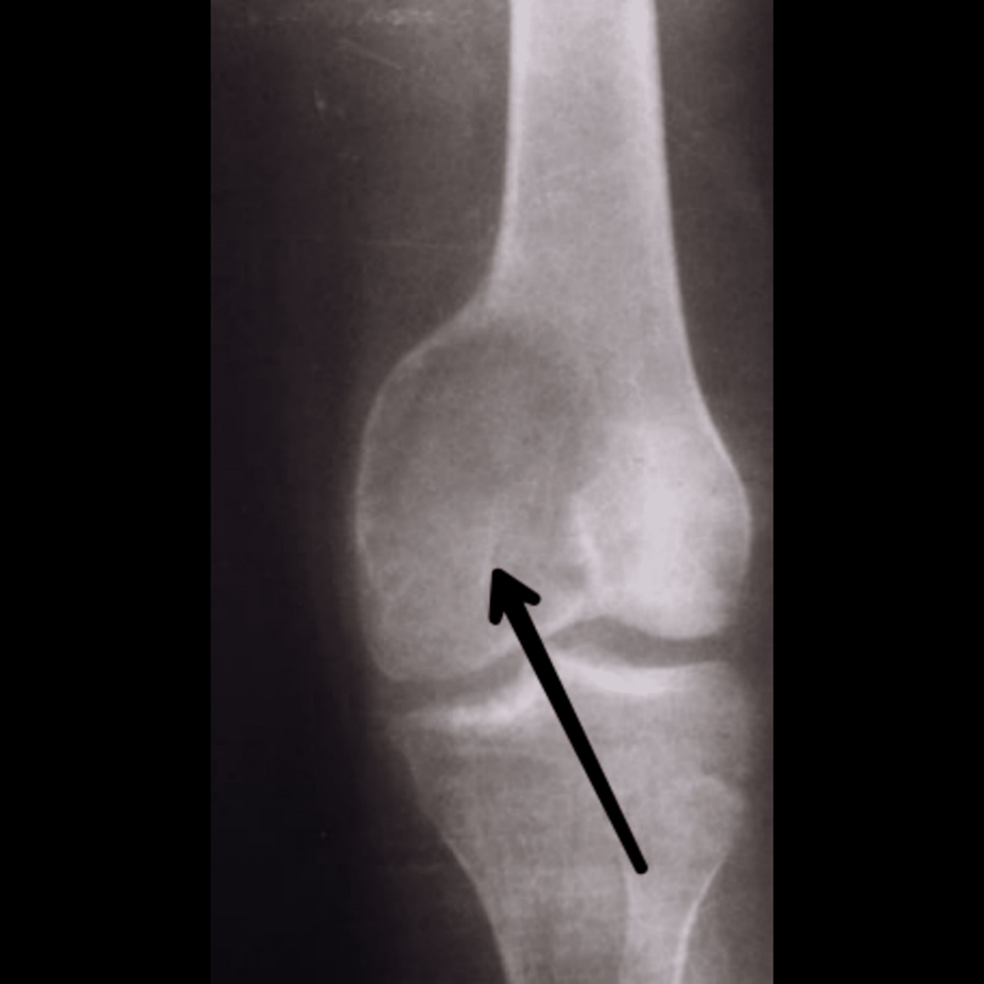
X-ray of knee joint showing GCTB in medial femoral condyle extending in the subarticular region. Image taken by Yash Jha. GCTB: giant cell tumour of bone

**Figure 4:**
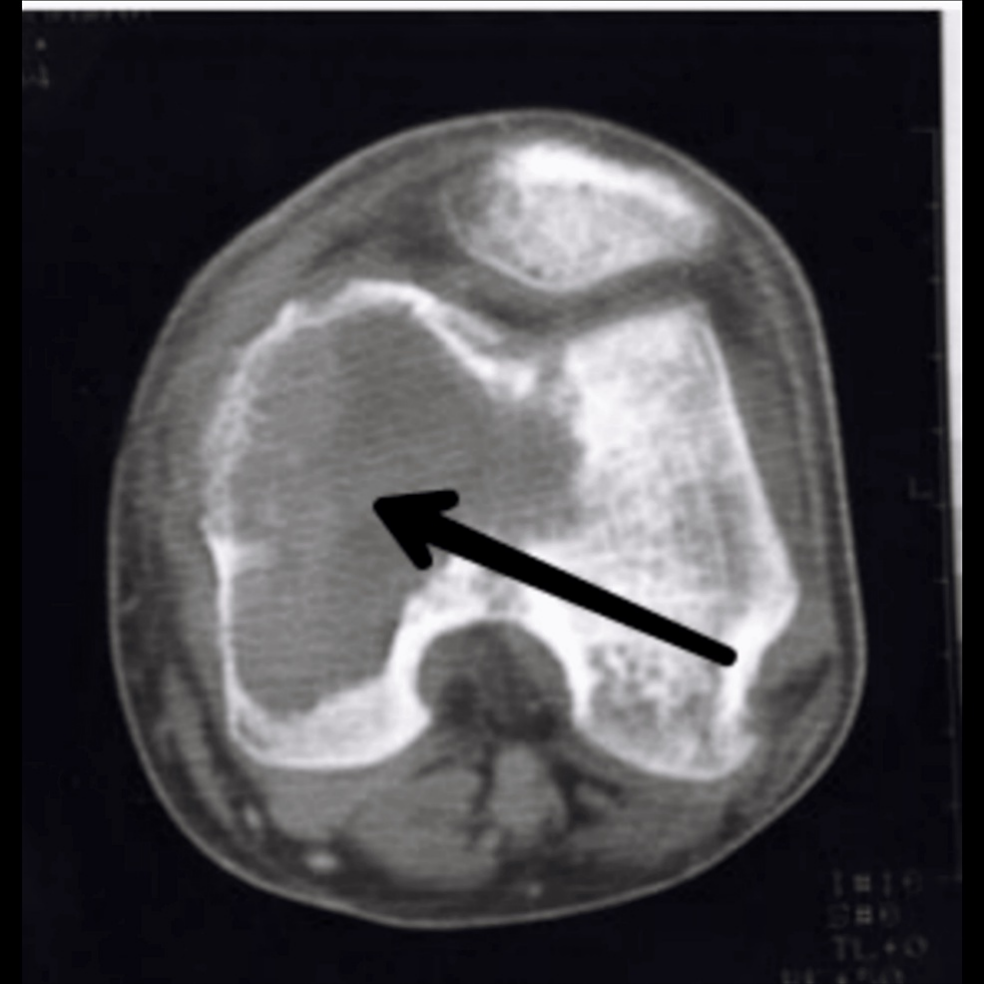
CT of knee joint showing GCTB in medial femoral condyle. Image taken by Yash Jha. GCTB: giant cell tumour of bone

Among all medical imaging techniques, MRI is the most crucial since it enables disease diagnosis, disease severity evaluation, and therapy planning. In up to 67% of cases, the preoperative MRI not only identifies the condition but also matches the postoperative findings [15]. For the staging and radiographic grading of the tumour, imaging tests are crucial. These tumours typically have radiological borders that are quite clearly defined, are clinically active, and are limited to the bone. The most prevalent illness is monocytosis. Even though GCT of the bone is related to enhanced vascularity, metastatic lung dissemination is uncommon. Extended intralesional curettage with or without adjuvant therapy is the predominant management strategy in surgery [1]. Although GCTB can frequently be suspected based on imaging with plain films, CT, or MRI, a biopsy is necessary for diagnosis. In many situations, the biopsy can also serve as the basis for definitive surgery. A tiny number of GCTB manifest as malignant GCTB with a more aggressive cytologic appearance, despite the great majority of GCTB having benign histologic characteristics. A pathologist with experience in this area should conduct the evaluation to rule out other diagnoses like giant cell-rich osteosarcoma, brown tumour of hyperparathyroidism, etc. because osteoclast-like giant cells can be present in many other conditions, including reactive conditions and other benign and malignant tumours. Both a non-contrast chest CT and an X-ray are necessary for staging [16]. Figure 1 shows an X-ray of a patient suffering from GCTB in the wrist joint. Figure 2 shows a CT scan of a patient suffering from GCTB in the wrist joint. Figure 3 shows an X-ray of a patient suffering from GCTB in the knee joint. Figure 4 shows a CT scan of a patient suffering from GCTB in the knee joint.

Diagnosis

Giant cells are found in many benign and malignant bone lesions, hence it is important to correlate clinical, imaging, and pathologic data when diagnosing GCTs in order to rule out other lesions with a similar histologic pattern [17]. In GCTB, the metaphysis and epiphysis frequently have lytic and eccentric lesions that extend to the articular cartilage. Trabeculation, which can be fine or coarse, is prevalent. The periosteal response is not prevalent, and the lesion's boundary is clearly defined and non-sclerotic. The cortex next to the GCTB is frequently punctured and thinned to varying degrees. Pathological fractures and kyphotic alterations are sometimes brought on by GCTs of the vertebrae. The lytic, non-mineralized character and absence of the periosteal response can be seen on CT. Findings from MRI are typically non-specific and comprise an increase in signal intensity on images obtained with fluid-sensitive sequences, a decrease in signal intensity on T1-weighted images, and enhancement following intravenous gadolinium contrast administration. Due to persistent haemorrhage and fluid-fluid level, intermediate to low signal intensity on T2-weighted imaging may be detected, suggesting aneurysmal alterations. Increased radiotracer uptake is seen on technetium 99m-methylene diphosphonate scintigraphy in the lesion's periphery, associated with central photopenia brought on by osteolysis or central necrosis (donut appearance) [18].

Treatment

At this time, proper surgery is the only curative treatment for GCTB. In general, the sort of surgery that would be suitable is determined by the Campanacci grade and the tumour's location (axial or appendicular). Curettage and adjuvant therapy are preferred for Campanacci grade 1 or 2 tumours (which do not extend beyond the cortex), but tumours with soft tissue extension (Campanacci grade 3) are treated with extensive local excision due to their high risk of recurrence [2]. Excision (curettage) is the most common surgical treatment, which tries to remove the tumour (intralesional resection) while preserving as much bone as possible. The removal of the entire tumour is the most important aspect of this treatment, and it should be done through a big enough cortical window to see the full defect. When planning surgery, MRI or CT imaging is useful for assessing the tumour's stage. The most popular surgical procedure, extended, intralesional curettage, has been proven to be efficient in the management of GCTB. It has been discovered that using bone cement to fill the osseous gap and, if necessary, placing an allograft on the subchondral bone surface are both efficient ways to reduce local recurrence and guard against articular cartilage damage. Steinmann pins or plate/screws (Zimmer Inc., Warsaw, USA) may be used when bone quality is a concern, particularly in situations when pathologic fractures are either imminent or have already occurred. However, their routine use is debatable [13]. Although radical resection is a surgical possibility in some circumstances, it is primarily used when there has been considerable osseous injury and significant bone loss [13]. In some cases with GCTB, medical care using either zoledronic acid or denosumab has shown promise as an efficient medical treatment. The role of medical care, in particular when to take these drugs (pre- or postoperatively), the length of treatment, and any potential dangers related to these drugs are still being assessed. For several years, local recurrence and metastatic spread surveillance should be maintained [13]. Denosumab treatment can stop GCTB from undergoing osteolysis, however, it cannot stop local recurrence. The clinical and pathological outcomes were nearly identical to those obtained prior to denosumab therapy, indicating that the changes in GCTB caused by this medication are reversible [19].

Risk

With the use of local adjuvant therapy, the high risk of recurrence associated with intralesional surgery can be reduced to about 30%. The Scandinavian Sarcoma Group demonstrated in their retrospective analysis that the likelihood of recurrence was decreased from 61 to 22% when cement rather than bone graft was used to fill the surgical void [2]. The second surgical approach is a wide resection that involves removing the affected bone fragment. Although this strategy significantly decreased the chance of local recurrence to only 0-12%, it may be overkill in some circumstances due to the severe morbidity it causes [2]. Recurrence of GCTB is observed in about 25% of patients, with curettage being associated with rates as high as 50%. It is uncommon for benign GCTB to occur simultaneously at several sites. Between 1% and 6% of benign tumours will spread, most frequently to the lung. Benign metastases can impair pulmonary function and occasionally be fatal, although typically being indolent [20]. However, repeat joint salvage can be used to treat 90% of local relapses following curettage. Since young adults make up the majority of GCTB patients, it is crucial to maximise joint salvage in order to maximise long-term function. With a higher likelihood of local relapse and a potential need for more severe therapy and tighter monitoring, younger patients and those with distal radius tumours treated with joint-sparing techniques should be particularly cautious [21]. Concern over elevated recurrence rates was raised as denosumab use became more common. However, the possibility of indication bias should be considered when interpreting meta-analysis results. A number of minor trials using short-course denosumab (ranging from three to six doses) have been published. With no statistically significant differences in radiological and clinical outcomes and a non-significantly increased recurrence rate following denosumab, a small trial directly compared denosumab with zoledronic acid [22]. Hypocalcemia, atypical femur fractures, and osteonecrosis of the jaw are some of the unfavourable consequences of denosumab. The question of treatment duration is significant because the latter two are linked to a time-dependent increase in risk, especially in the substantially younger population normally affected by GCTB than by post-menopausal osteoporosis or carcinomatous metastases [23].

Histology

GCTB has a friable look and a dark brown to reddish colour. The histological appearance of GCTB traditionally exhibits a stroma of mononuclear spindle cells and monocytes in the foreground and several big multinucleated giant cells in the background [13]. Neoplastic cells (osteoclast precursor and spindle-shaped stromal cells) and reactive cells, such as sizable, multinucleated giant cells that resemble osteoclasts, make up GCTB [20]. In spite of their benign outward appearance, GCTs of the skeletal system are thought to be aggressive and have the most unpredictable neoplasms. Histology or flow cytometry is not yet a reliable means of predicting distant metastases in GCT [24]. Histologic characteristics of cancer in GCTB are:

a. Primary malignant GCTB, pre-denosumab: Undifferentiated pleomorphic sarcoma is indicated by the proliferation of atypical spindle and pleomorphic cells developing in fascicles; GCTB is indicated by the proliferation of ovoid to spindle bland-appearing cells with scattered reactive multinucleated osteoclast-like large cells.

b. Secondary malignant GCTB, pre-denosumab: GCTB-like histological characteristics coexisted with an unusual spindle cell proliferation that invaded between the host bone trabeculae, which was a sign of a high-grade, undifferentiated spindle cell sarcoma [25].

According to available data, benign GCT of bone has three different cell types:

Type I cells have the ability to multiply, resemble interstitial fibroblasts, and produce collagen. The tumour component of GCT is probably this cell population. The mesenchymal stem cells from which type I cells may have been formed share some characteristics with type I cells; however, type I cells also have characteristics that may indicate an early differentiation of mesenchymal stem cells into osteoblasts.

Type II cells can be recruited from the peripheral bloodstream and are also interstitial, but they resemble the monocyte/macrophage family. By fusing with one another or by nuclear multiplication without cell division, these cells are regarded to be the forerunners of the multinucleated giant cells. Giant cells lack the monocyte/macrophage lineage surface receptors that are expressed by type II cells.

The multinucleated large cells are type III cells. They morphologically resemble osteoclasts and share many of their traits. They have bone resorption enzymes such as type II carbonic anhydrase and tartrate-resistant acid phosphatase. The majority of the unique osteoclast antigens are shown by GCT large cells. There are calcitonin and vitronectin cell receptors, and matrix metalloproteinases (MMPs) are expressed [26].

Tumour markers

Serum acid phosphatase has been studied for its potential utility in GCT as a tumour marker. According to one study, half of the cases had high acid phosphatase levels, while the other half had levels that were normal. Acid phosphatase concentrations were higher in those with larger GCT lesions. However, postoperatively, serum acid phosphatase levels significantly dropped in both populations. In spite of these findings, acid phosphatase is not a reliable screening indicator for sufficient resection or local recurrence. The tumour marker creatine kinase isoenzyme BB (CK-BB) is also being studied in GCT. In order to regenerate adenosine triphosphate, this enzyme catalyses the reversible transfer of phosphate from phosphocreatine to adenosine phosphate. It is hardly ever discovered in the serum of healthy people. Patients with brain traumas, stomach cancer, prostate cancer, and particular forms of osteopetrosis have seen it most frequently. Patients with osteosarcoma, aneurysmal bone cysts, malignant fibrous histiocytomas, or common osteolytic entities do not have increased levels. Patients with GCT have high CK-BB levels at the time of diagnosis, and two weeks following excision, their CK-BB levels recover to normal. In spite of these findings, CK-BB does not serve as a reliable screening indicator for sufficient resection or local recurrence [27].

Latest advancement

Denosumab may be administered as neoadjuvant chemotherapy in GCTB patients where surgical resection is the preferred main treatment option in order to facilitate tumour excision. To achieve a greater tumour resection margin, pre-operative denosumab is administered to inhibit tumour osteolysis, raise bone mineral density, increase marginal sclerosis, and reconstitute the articular surface [28]. Additionally, it can shrink the tumour and form a calcified rim around the soft tissue component of mature GCTB, enabling curettage with local adjuvants at a later stage in previously unresectable GCTB [29]. There is no information on how long individuals who have taken neoadjuvant denosumab should continue taking this medicine in the adjuvant situation. Patients got six doses of denosumab following surgery in the registration trial for the drug in GCTB [30].

## Conclusions

The article provides more clarity about GCTB which is a benign aggressive tumour. The Campanacci grading method is the most frequently mentioned classification method. Major imaging technique like plain radiography, MRI and CT role in the diagnosis of GCTB has also been explained. Due to the development of these imaging techniques, it has become easy for doctors to diagnose GCTB. The article also explained different treatment procedures that are required to be followed based on the Campanacci grading system and the tumour's location. The risk factors involved in its treatment and chances of recurrence after treatment have also been assessed. The role of denosumab is also explained. Types of cells found in benign GCTB and histological characteristics of cancer in GCTB have also been discussed in this article.
